# The Association between Proximity to Animal Feeding Operations and Community Health: A Systematic Review

**DOI:** 10.1371/journal.pone.0009530

**Published:** 2010-03-10

**Authors:** Annette M. O'Connor, Brent Auvermann, Danelle Bickett-Weddle, Steve Kirkhorn, Jan M. Sargeant, Alejandro Ramirez, Susanna G. Von Essen

**Affiliations:** 1 Department of Veterinary Diagnostic and Production Animal Medicine, College of Veterinary Medicine, Iowa State University, Ames, Iowa, United States of America; 2 Texas AgriLife Research, Amarillo, Texas, United States of America; 3 Center For Food Security/Public Health, College of Veterinary Medicine, Iowa State University, Ames, Iowa, United States of America; 4 National Farm Medicine Center, Marshfield Clinic, Marshfield, Wisconsin, United States of America; 5 Centre for Public Health and Zoonoses, and Department of Population Medicine, Ontario Veterinary College, University of Guelph, Guelph, Ontario, Canada; 6 Department of Environmental, Agricultural and Occupational Health, College of Public Health, University of Nebraska Medical Center, Omaha, Nebraska, United States of America; The Kenya Medical Research Institute, Kenya

## Abstract

**Background:**

A systematic review was conducted for the association between animal feeding operations (AFOs) and the health of individuals living near AFOs.

**Methodology/Principal Findings:**

The review was restricted to studies reporting respiratory, gastrointestinal and mental health outcomes in individuals living near AFOs in North America, European Union, United Kingdom, and Scandinavia. From June to September 2008 searches were conducted in PUBMED, CAB, Web-of-Science, and Agricola with no restrictions. Hand searching of narrative reviews was also used. Two reviewers independently evaluated the role of chance, confounding, information, selection and analytic bias on the study outcome. Nine relevant studies were identified. The studies were heterogeneous with respect to outcomes and exposures assessed. Few studies reported an association between surrogate clinical outcomes and AFO proximity. A negative association was reported when odor was the measure of exposure to AFOs and self-reported disease, the measure of outcome. There was evidence of an association between self-reported disease and proximity to AFO in individuals annoyed by AFO odor.

**Conclusions/Significance:**

There was inconsistent evidence of a weak association between self-reported disease in people with allergies or familial history of allergies. No consistent dose response relationship between exposure and disease was observable.

## Introduction

Livestock and poultry operations that feed large numbers of animals are common in the USA. Facility capacity varies greatly by region and it is not uncommon for barns to house 1,000 swine with multiple barns at a single site, feedlots to house 50,000 cattle, and poultry houses to house 100,000 hens. Facilities with a large number of animals are frequently referred to as concentrated animal feeding operations (CAFO) [1]. There is primary research suggesting that livestock facilities that confine animals indoors for feeding can represent an occupational hazard for workers [2]–[5]. The health effects are primarily associated with respiratory system function [2]–[5]. Several primary research studies have also investigated whether these adverse health effects spill over into the communities of individuals living near animal feeding operations. Several narrative reviews have attempted to summarize the association; however none of the reviews available applied the systematic review methodology to the topic area [6]–[14].

The systematic review methodology has been applied as the method of summarizing the scientific information about a topic to many areas in the clinical sciences, social sciences, food safety regulation and environmental sciences [15]–[17]. The methodology has also been recommended and applied to the evaluation of epidemiological studies to assess environmental risk [18]–[20]. The systematic review methodology has several key principles: transparency, comprehensiveness and evaluation of the primary research study design. Transparency refers to the reporting of all aspects of the review to enable the reader to assess the validity of the review process and potential biases. Comprehensiveness refers to a broad, clearly described approach used to identify the literature to be considered for the review. Finally, the systematic review methodology evaluates the primary research for the presence of study design features, identified by content and methodological experts, necessary to make the primary research valid for the review question.

The product of the systematic review methodology depends upon the quantity and quality of the primary research ultimately available. If sufficient high quality primary research is available, a summary effect measure may be calculated, i.e., a weighted average of effects across studies which may be a better descriptor of the expected outcome than data from any single study. This approach is usually limited to studies of interventions with homogenous outcomes. If the quality of primary research is poor, the quantity sparse, or the outcomes heterogeneous, then calculation of a summary effect may not be possible or sensible. When calculation of a summary effect is not possible, as is often the case when epidemiological studies are used, the review may summarize the results of the relevant studies and highlight deficiencies in the quantity, focus, design, analysis, or reporting of the primary research [21].

The purpose of this review was to apply the key aspects of a systematic review methodology to address the question “What is the association between animal feeding operations and the measures of the health of individuals living near animal feeding operations but not actively engaged in livestock production in North America, the European Union, the United Kingdom, and Scandinavian countries?”

## Methods

### Review Protocol and Panel Qualifications

The approach to conducting the review was guided by the World Health Organization (WHO) report “Evaluation and use of epidemiological evidence for the environmental health risk assessment” [18]. Working protocol forms were obtained from the Collaboration for Environmental Evidence [22]. The approach to reporting the review was guided by the QUORUM statement with modification for observational studies [23].

The first author (AOC) responded to a solicitation to conduct a review funded by the United Soybean Board in 2008 and was the review manager. Five individuals, who had previously authored reviews or primary research about animal feeding operations and community health impacts, with experience in one of the above areas were approached to participate in the review and three accepted the invitation [7], [9], [12], [14], [23]. Four individuals who were not authors of reviews or primary research in the topic area but had training and experience in either public health or epidemiology were approached to participate and three agreed. The final seven reviewers included two veterinarians' with doctoral degrees in epidemiology, an occupational health physician with a masters degree in public health, pulmonologist with expertise in occupational lung disease with a masters degree in public health, one veterinarian with a masters degree in public health, one veterinarian with a doctoral degree in microbiology, and an agricultural engineer with a doctoral degree in chemical and bioresource engineering. Five reviewers were familiar with systematic reviews; two reviewers had managed a systematic review and one had been a reviewer on a systematic review.

The reviewers were first convened by conference call in July 2008 and the working protocol derived from the original proposal. The protocol was a working document, which was referred to and updated as needed during the review.

### Selection

The review question agreed upon was: What is the association between animal feeding operations and the measures of the health of individuals living near animal feeding operations but not actively engaged in livestock production in North America, the European Union, the United Kingdom, and Scandinavian countries? The following definitions were also agreed upon:

1. Animal feeding operations: Facilities (pasture/buildings) used to house animals for food production on any scale.
2. Health: Health is a state of complete physical, mental, and social well -being and not merely the absence of disease or infirmity as defined by WHO.
3. Actively engaged: Owning or working on a livestock production facility

Relevant studies were primary research studies reporting the respiratory, gastrointestinal and mental health outcomes measured directly on human subjects. The population of interest was communities living near livestock production in North America, the European Union, the United Kingdom, and Scandinavian countries as the production systems in these areas are most relevant to the target audience. An exact distance for “near” was not defined as some reviewer members suggested correctly that the majority of manuscripts would not include this information and this would exclude many papers from the review. The reviewers did not limit the review to research that met the U.S. Environmental Protection Agency definition of concentrated animal feeding operations (CAFO) because again experience suggested that many papers would not provide sufficient information to clarify the livestock population size [1]. For this reason the operations are generically referred to as animal feeding operations (AFOs). The review was also restricted to publically available peer-reviewed literature.

### Searching

The search was designed to be comprehensive and used electronic searches, hand searching and personal contacts for the identification of literature. Electronic searches were conducted for three health outcomes: including respiratory disease (conducted in June 2008), gastrointestinal disease (conducted in September 2008), and mental health outcomes (conducted in September 2008) in PUBMED, CAB, Web of Science, and Agricola from inception dates. After adjusting for minor differences in syntax rules between the databases, each search had similar components with key words included to identify: (animal feeding operations) AND (community) AND (health/disease). For example, the animal feeding operation search string used in PUBMED was (cafo* OR “animal feeding operation” OR “livestock operation” OR “large-scale swine operation” OR “large-scale hog farm” OR feedlot OR “confined swine feeding” OR “industrial hog operations” OR piggeries OR sties OR confinement OR “animal housing” OR “livestock facilities” OR “Industrial hog farming operations” OR “intensive livestock” OR “farm exposures”). The community string was (community health” OR “school health” OR “neighbor health” OR “environmental health” OR “public health”). The health outcome terms used for gastrointestinal disease were (diarrhea) AND (North American, Europe) NOT (BVD, BVDV, Scours). The gastrointestinal disease string included a NOT term to limit the number of papers that reported gastrointestinal disease in animals as the health outcome and non-target regions.

Time limits or language restrictions were not imposed upon the searches. For each health outcome, citations from the four databases were combined into a master database and duplicates excluded based on parameters of same author, date, and title. The master database for each clinical term was used for first level relevance screening (see below).

Hand searching was also used to identify the literature. After identifying relevant manuscripts (see below), their reference lists were checked to identify manuscripts not present in the master database and, if not found, these were retrieved and added to the manuscript relevance screening process. In addition, the reference list of narrative reviews about community health and AFOs [6]–[14], [24]–[41] were checked to identify manuscripts not in the master database and, if not found, these were added to the manuscript relevance screening process. Further, if the electronic searches and hand checking identified non-peer reviewed publications such as theses or conference proceedings, a first author search was conducted in PUBMED, CAB, Web of Science, and Agricola with no date or language restrictions. Peer reviewed publications identified by the first author search were included in the relevance screening process.

Several personal contacts resulted in identification of manuscripts to be considered for the review. Four panel members suggested particular papers and we became aware of a group conducting a similar review in Quebec and that group kindly shared the review reference list (pers. comm. G. Brisson). Publications identified this way were included in the review and a first author search was conducted in PUBMED, CAB, Web of Science, and Agricola. Potentially relevant publications were included in the relevance screening process.

### Relevance Screening

The purpose of the manuscript relevance screening was to rapidly remove articles not relevant to the review. Two levels of relevance screening were used. Staff members in the first author's research group (AOC) conducted all relevance screening. These staff members were familiar with the relevance screening process in systematic reviews after having participated in several reviews. Two reviewers independently evaluated each citation using the first relevance-screening question, “Does the title and/or abstract describe primary research reporting the association between livestock and human interactions (direct or indirect) and measures of human health measured on humans.” The 1^st^ level relevance screening reviewers were not masked to the author or journal source. Citations were only excluded if both reviewers responded “no.” Titles and abstracts not written in English were excluded. Non-English papers with English titles and abstracts were included in relevance screening.

For citations that passed the 1^st^ relevance screening, the 2^nd^ relevance-screening question applied was, “Does the title and/or abstract describe primary research reporting the association between livestock and indirect human interactions (i.e., in the community, not employees or farmers) and measures of human health measured on humans.” The process for assessing and passing citations through 2^nd^ level screening was the same as first relevance screening; however the first author (AOC) was always one of the reviewers for the second relevance screening. The 2^nd^ level relevance screening reviewers were not masked to the author or journal source.

### Validity Assessment and Data Abstraction

After relevance screening, the full manuscripts were obtained and distributed to the review panel members. An Iowa State University employee in the foreign languages department translated non-English manuscripts including tables but not reference lists.

Manuscripts were allocated to reviewers using a blocked random number generator, ensuring each reviewer received the same number of manuscripts and each manuscript had two reviewers. The only exceptions to this approach were two German language papers [42], [43]. One reviewer was a native German-speaker, and therefore this reviewer was assigned the German language papers. A translated copy was assigned to the other reviewers. All panel members, except the first author (AOC), were masked to the title, author and journal information by blacking out all title, author, and journal information on the PDF file. However, some manuscripts were well known by some reviewers and likely recognizable. For each manuscript, panel members were first asked the 2^nd^ relevance-screening question. If both reviewers responded no, the paper was not considered relevant and not reviewed further.

All reviewers then independently extracted the following information and returned the extracted information to the panel manager.

1. What is the time frame the study was conducted?
2. What is the location of the study population?
3. What is the study location area?
4. What is the size of the human population under study?
5. What is the size of the animal population under study?
6. What is the unit of concern?
7. What is the study design?
8. What is the definition of an “exposed” person or community?
9. What is the definition of an “unexposed” person or community?
10. What is the health outcome measured?
11. What are the “animal” variables used?
12. What statistical approach is used to assess the association?
13. What measure of association is reported?
  In accordance with the recommendations of the WHO guidelines, the reviewers were also asked to independently respond to the following questions [18].
14. Is the study question clear?
15. Is the exposure assessed using valid and reliable measures?
16. Is the health outcome(s) assessed using valid and reliable measures?
17. Is the study design appropriate?
18. What approach or analysis of the data is used to take into consideration: *chance, confounding, information bias, selection bias and analytic bias?*

Definitions of chance, confounding, information bias, selection bias and analytic bias were provided [18].

### Qualitative Data Synthesis

After the reviews were conducted and compiled, a consensus meeting was held in February 2009. The meeting allowed all reviewers to discuss the manuscripts and vote which should be included in the final summation of evidence due to evidence of substantial bias that may have affected the internal validity of the study results.

Prior to the meeting, panel members received a draft of the review including comments about bias for each manuscript from review evaluations, the extracted data and a copy of each review manuscript (unblinded). At the meeting, a reviewer presented a synopsis of their responses to questions 1–18. After discussion, a silent vote was taken with each panel member indicating if he/she felt confounding, chance, information bias, selection bias and analytical bias should be discussed in the review summation. The criterion was that the reviewer felt the bias was substantive, i.e., potentially resulting in a meaningful difference in the inference. For manuscripts that received a majority vote (4/7) this was noted in the results and discussion.

### Data Extraction

For relevant studies the following data extraction rules were used:

1. Only results from multivariable analysis, which adjusted for important known confounders of the outcome, were extracted unless otherwise noted. If authors used terms such as “adjusted for,” “considered as potential risk factors,” “evaluated confounders,” or “adjustment of covariates,” it was assumed a multivariable approach was used, as the model building process was rarely described.
2. When multiple modeling approaches were reported for the same main group, the results from the model that corresponded to that reported in the abstract were extracted.
3. Main-group analysis results were extracted in preference to subgroup analysis unless a significant interaction was reported, then the results from each level of the effect modifier were extracted.
4. When the results were presented as a beta (β) coefficient from a regression model, the β was extracted and not converted to an effect measure.
5. When point estimates and standard errors were reported, 95% confidence intervals were calculated from these data.
6. If the data were reported as only a graph, attempts were made to extracted data from the graph.
7. All reported dependent to independent variable associations from regression models were extracted. For example, when a manuscript reported the odds ratio and 95% confidence interval for three dummy variables describing the association between a binary dependent variable and an independent variable with four levels, all three of the odds ratios and 95% confidence intervals were extracted and referred to as an observation. No authors reported the p value for the main effects.

## Results

4,908 articles were retrieved from the search process. After the relevance screening process 28 manuscripts were identified as potentially relevant and distributed for further evaluation. During the independent review, seven more manuscripts were identified as not relevant because they either evaluated occupational hazards or did not include a control group [44]–[50]. After full review, ten studies, including nine ecological and one case control, were deemed not relevant to the review as it was not possible to differentiate between occupational versus community cases of disease [51]–[60]. Finally a series of publications about the same study were identified, and the results from the two smaller earlier reports were not included [43], [61] in favor of the final publication that included the largest population [62]. Nine studies were considered relevant to the review (TABLE 1).

**Table 1:** Summary information for studies.

Author	Study design	Country	Number of subjects eligible for analysis *	Age of subjects	Method of analysis
[42]	Cross-sectional	Germany	3867	5-to 6 year old	Multivariable logistic regression with fixed effects only.
[68]	Cross-sectional	USA	47651	Middle-school age	Multivariable logistic regression with fixed and random effects. Specifically a 2-level random-intercepts model, with binary outcome variable. One level was student level factors the other school level factors.
[62]	Cross-sectional	Germany	1855 (symptoms) 810 (lung function)	Adults	For self-reported symptom outcomes a multivariate logistic regression with fixed effects. For differences in lung function parameters a multivariate linear regression with fixed effects only.
[69]	Longitudinal	USA	15	Adult	MultivariableMultivariate linear hierarchical mixed models. Two levels were modeled: day (within person) and person (within cluster).
[65]	–Cross-over (experimental)	USA	48	Adult	An analysis of variance was performed to determine if there were any main effects or interactions between group (control or experimental) and gender for each Profile of Mood Status factor and the total mood disturbance. Subjects were nested within group and gender.
[66]	Cross-sectional	USA	309	Elementary school children	MultivariableMultivariate logistic analysis with fixed effects only. Exposure described as attending a school near an AFO measured at the group level (n = 2).
[67]	Cross-sectional	USA	155	Adults	MultivariableMultivariate linear regression with exposure defined a living near a particular swine or cattle AFO group level
[63]	Cross-sectional	USA	82	Adults	UnivariableUnivariate analysis
[64]	Cross-sectional	USA	36	Adults	UnivariableUnivariate analysis

*Studies rarely reported the missing data or methods for handling missing data in the analysis, therefore all study subjects eligible may not have been included in analysis.

### Evaluating Sources of Biases

#### Confounding bias

Of the nine relevant studies two observational studies did not adjust for any potential confounders [63], [64]. The results of these studies were not extracted. One study did not appear to adjust for covariates however the investigators allocated the study subjects to exposure in a crossover experimental model and incorporated an effect for non-independence of repeated study participant observations [65]. The results of this study were extracted.

The remaining studies used multivariable methods to control for potential confounders associated with the outcomes. However the potential for confounding was considered to be high for two studies that used designs with cluster/site level indicators of a risk factor represented by a single observation. [66], [67]. For example, Sigurdarson reported a cross sectional study where the measure of the exposure to AFOs was the location of the elementary school with respect to an AFO [66]. A binary outcome (asthma, yes/no) was measured on individual students at the school. However, the study included only one school for each level of the exposure variable, i.e., one school in Northeast Iowa 0.5 miles from a facility that housed 3800 hogs and one control school with no facility within 10 miles. The school-level exposure variable was not measured at the same hierarchical level as the outcome, asthma in the individual child, and therefore no variation within site was possible. The results were potentially confounded by other factors associated with each school, such as the condition of the school building and the presence of other local industries, and the outcome, asthma. Another study with a similar design did not suffer from this potential issue by incorporating data from students at over 200 schools and ensuring variation in the exposure measure [68].

A similar approach to the research question was used to assess the association of health in individuals living near a swine or cattle AFO [67]. The explanatory variable used to measure exposure to AFOs was whether the study subjects were residents of three rural communities, one in the vicinity of an approximately 6,000-head hog operation, one in the vicinity of two intensive cattle operations, and a third rural agricultural area without livestock operations that use liquid waste management systems. However, only one observation for each level of the explanatory variable was measured including one community close to a swine operation, one community close to two cattle operations, and one control community.

#### Multiple comparisons/chance

Many studies assessed multiple variables and outcomes and therefore the impact of multiple comparisons is a concern in this general area of research. For example one study reported 32 unique beta estimates from a linear regression based on data from 155 respondents [67] and another study reported 36 p-values from an experimental study with 48 participants allocated to two exposure types [65] suggesting the potential for a high family-wise error rate. Other studies also reported multiple comparisons however the study populations were considerably larger (Table 1). No studies reported adjustment for multiple comparisons.

#### Misclassification bias

Retrospective self-reported case definitions that may be subject to information bias (misclassification) and awareness bias represent a potential of bias in all but two studies [65], [69]. Further the accuracy of the definitions of exposure to livestock could not be determined and were therefore extremely difficult to critique and no study had a clearly better definition of exposure to animal feeding operations compared to others.

#### Analytical bias

It is very difficult to assess analytical bias as statistical methods are frequently described poorly in many areas of research [70]. Further it is unclear if the apparent biases are due to true errors in analysis or omissions in the descriptions. For example, one group of authors reported using linear regression for data with only four possible outcomes: never, occasionally, sometimes, or often [67]. This ordinal outcome did not appear to be continuous as required by a linear model and suggested the possibility of analytical bias. A discussion of model fit, which may have alleviated these concerns, was not included in the methods section. Additionally, the betas from the linear model appeared to be interpreted as a ratio, “*Only episodes of excessive coughing and heart burn occurred on average >2 times more in the cattle than in the control community (β>2)*”, where the standard interpretation for a beta from a linear model would be a one unit increase in the outcome for a one unit increase in the explanatory variable, i.e. two more episodes of disease. These discrepancies lead to concerns about the potential for analytical bias to influence the outcome of the study. Another study was also identified as having the potential for analytical bias [64]. Individuals in the study were treated as independent despite the presence of households as clusters. Further the manuscript reported using a Wilcoxon test for the analysis of a categorical outcome, yet reported T values in the tables of results [64]. Finally, the authors report a one-tailed test with 26.7 degrees of freedom. Decimal points are not commonly used for degrees of freedom of univariable analyses.

#### Selection bias

Selection bias refers to the differential enrollment of one study group compared to other groups, i.e. different selection odds, and selection bias is difficult to assess and can only be suspected rather than proven. One study reported the case group selection methods as followed “*respondents who lived near industrial hog farms and had been identified by local grass-roots activists as individuals who were distressed about the effects of the nearby hog farms.”* [63]. This approach seemed likely to increase the selection odds of the disease and exposed groups relative to other study groups. This study reported a decreased perceived control in study participants who lived close to an AFO.

Another study reported recruiting 18 of 27 neighbors who lived within two miles of a 4,000 head sow unit, nine households with 18 study subjects completed the study indicating a 30% participation rate among those approached to be in the case group [64]. For the control group, 188 rural residents (assumed to be households) from a county with low animal density based on 1992 agriculture census data were approached to enroll. Eleven households with 21 individuals agreed to participated, however only nine households with 18 participants were eventually enrolled due to eligibility criteria, indicating a participation rate of 5%. The differences in participation rate for the study groups may indicate a high potential for selection bias.

Other studies also had the potential for selection bias due to the failure to use random selection methods for study participants increasing the likelihood of selection bias [68].

#### Data extraction and summarization

Data were extracted from five studies that used multivariable analyses and used more than a single observation for the evaluation of the risk factor [42], [62], [65], [68], [69]. For these studies, the purpose of the study, the outcome assessed (disease symptom or lung function), and methods of measuring the exposure variable of interest (exposure to AFO) are included in TABLE 2. Self-reported outcomes such as wheeze, asthma, and depression were commonly used [42], [62], [68]. A variety of standard questionnaires and subsequent definitions were used to capture self-reported outcomes, including: The International Study of Asthma and Allergies in Childhood (ISAAC) [42], [68], the short form 12 Health Survey [43], [62], The Environmental Exposures and Health questionnaire [65] and The Profile of Moods States (POMS) scale [65]. Other studies used lung function parameters as measures of the outcome, such as percent change in forced expiratory volume at one second (FEV_1_), percent change in forced vital capacity (FVC), and percent change in forced expiratory flow between the full expiration of 25 and 75% of the total FVC (FEF 25–75%) [65]. Other studies used clinical indicators of the outcomes such as bronchial hyper-responsiveness to methacholine [62], specific IgE to common allergens (IU/ml) [62], specific IgE to agricultural allergens (IU/ml) [62], salivary IgA (ug/ml) [65], pg/ml of the proinflammatory cytokines interleukin (IL)-1β and IL-8 in nasal lavage [65], cell counts [65], percent and absolute epithelial cells [65], percent lymphocytic cells [65], and percent polymorphonuclear cells [65].

**Table 2:** Descriptive characteristics of the final review studies.

Study	Human reference population and outcome assessment	Animal reference population and animal exposure assessment
[42]	School children in a region of Lower Saxony with intensive agriculture (counties of Cloppenburg, Emsland, Oldenburg and Vechta). (Of GERMANY )Parents were asked about asthmatic and allergic symptoms of their (generally) 5 to 6 year old children, as well as about possible risk factors.	For the exposure determination, databases from the Lower Saxony counties of Cloppenberg, Emsland and Vechta were available with a total of about 12,000 registered animal stalls, including information about the geographical coordinates of the stalls, the kind of animal being held there (cattle, swine, poultry, turkeys) and the size of the herds. The emission strength of bioaerosols for each stall was calculated based on the kind of animal, size of the herd, and published emission factors … The geographical coordinates of the homes and thereby the relative position in relation to the animal barn were determined from the home address of the subject. The exposure of the subjects was thus calculated from the sum of the individual bioaerosol emission contributions of the surrounding animal stalls (in a radius of 2km) on the particular home* translated from original text in German
[62]	The study was conducted in 4 rural towns in Lower Saxony, northwestern Germany, with a high density of animal feeding operations (Table S1). The animal production focused primarily on pigs and poultry. All adults age 18 to 44 years with German citizenship, registered in the population (n = 10,252). The registry provided information on home addresses, age, and sex of the target population.	Exposure to confined animal feeding operations was defined by the self-reported level of odor annoyance in the home environment (“How annoyed are you by odor in and around your home?”). The question on odor annoyance was assessed on a 4-point Likert scale from “not at all” to “strongly.” Ninety percent of subjects reporting to be at least somewhat annoyed by odors in the home environment reported the agricultural sources (spraying of the fields, confined animal feeding operations) were the major source of odor. Separate exposure estimates were developed on the basis of number of animal houses within 500m (0.3 miles) around participants' home. The distance was chosen because microbial emissions can be measured up to 500m from confined animal feeding operations.
[68]	Middle school aged children North Carolina. Students completed questions from the International Study of Asthma and Allergies in Childhood questionnaire, a standardized and validated instrument that combines a traditional written questionnaire with a series of video scenes that show children with asthma symptoms.	Estimates of exposure to airborne pollution from 2343 swine CAFOs were generated using data from permits that were issued by the North Carolina Division of Water Quality to all CAFOs that house at least 250 animals and use a liquid waste management system…. Separate exposure estimates were developed on the basis of distances between schools and swine CAFOs and of survey responses about noticeable odors from livestock farms. Distances and geographic directions between schools and CAFOs were calculated using the formulas given by Goldberg et al and Sinnott, respectively. We used calculations of proximity to create 3 metrics of potential exposure for each school: (1) distance to the nearest operation; (2) SSLW within 3 miles; and (3) a weighted SSLW based on the distance between the school and nearby swine CAFOs, the SSLW of each operation, and the proportion of wind measurements in the direction from the operation to the school. We obtained measurements of wind speed and direction recorded at 16 automated weather stations located throughout the state from the State Climate Office of North Carolina (Raleigh, NC).
[69]	The total N was 15 participants. One representative of each household cluster was selected. In conjunction with local community organizations, we identified exposed communities and recruited study participants in five geographic clusters. Participants were nonsmoking adults who lived within 2.4 km (1.5 mi) of an intensive hog operation and had at least one neighbor within 0.4 km (0.25 mi) of their home who was also willing to participate. One person from each household participated. Clusters included two to four households. Participants in each cluster agreed on two times, approximately 12 hr apart (for example, 7:00 AM and 7:00 PM), at which they would collect data for 14 days	Three clusters were near a single hog operation, one was near two hog operations, and one was near four operations. The permitted number of animals in each operation ranged from 1,000 to 12,000.
[65]	Forty-four experimental (persons living near hog operations) and 44 control subjects participated in the study; all of the subjects were residents of North Carolina. The subjects in the two groups (control and experimental) were matched according to gender, race, age, and years of education…. Mood ratings were obtained from all subjects by filling out Profile of Mood States questionnaires (POMS). The POMS was chosen to measure the impact of the hog odors on mood because it has been shown to be sensitive to transient mood shifts [65], [66]. There are 65 adjectives/feelings on the POMS, most of which may be grouped into one of six factors: tension/anxiety, depression/dejection, anger, hostility, vigor/activity, fatigue/inertia, and contusion/bewilderment. Each feeling is rated on a scale from 0 (not at all) to 4 (extremely).The feelings for each factor were added together, according to the POMS manual, to get a total score for that factor. The totals for each factor were then added together, with the vigor/activity factor weighted negatively, to derive a total mood disturbance score.	Experimental subjects were asked to complete one POMS questionnaire per day on 4 days when the hog odor could be smelled. The 4 days did not have to be consecutive, and subjects had as long as needed to complete all four POMS questionnaires. Control subjects were asked to complete one POMS per day for 2 days. All subjects were asked to complete the POMS based upon how they recently had been feeling, including at that particular time.

There was no homogeneity with respect to measures of exposure to AFOs or the health outcomes, making it unreasonable to conduct a meta-analysis of the reported associations. One study was unique in that study subjects were purposely exposed to a diluted air sample from a swine confinement in a cross-over control experimental model [65]. The association between the outcomes and the exposure group formed the basis for assessment. Other studies used indirect measures of exposure such as the number of hog pounds or number of AFOs in the vicinity [62], [68], [69]. One study estimated endotoxins in the area as a measure of exposure [65]. Several studies used detection of odor or odor annoyance as a measure of exposure [62], [68].

The results from all adjusted associations between surrogate clinical outcomes and the measures variable of proximity to AFO interest are presented in TABLE 3. Study participants with greater than 12 animal houses within 500 meters of their house were associated with a significant decrease in % predicted FEV_1_ (adjusted means difference in % predicted –FEV_1_ = −7.4, 95% CI −14.4 to −0.4) [62]. Other associations between exposure to diluted air from a swine confinement facility and percent epithelial cells and percent lymphocytic cells were only reported as p values rather than effect measures, so the magnitude of association was not available. The remainder of associations did not indicate strong associations as the point estimates were close to the null value and confidence intervals were wide. Further, for ordinal explanatory variables, the point estimates switched from above to below the null value as the implied risk of exposure increased, failing to provide evidence of an association that was not detected by hypothesis testing (N.B. all authors of the primary research papers used significance testing with p<0.05 as the criteria for significance).

**Table 3:** The reported adjusted* association between clinical outcome variables and measures of proximity to AFOS.

Study	Outcome variable	Community health/animal exposure measure	Subcategory	Effect measure	Point estimate	95% CI point estimate
[62]	Specific IgE to Common Allergens >0.35 IU/mL	How annoyed are you by odor in and around you home?	Not at all	OR	1.00	
			Somewhat	OR	1.11	0.79–1.57
			Moderately	OR	1.71	1.02–2.87
			Strongly	OR	1.02	0.51–2.03
[62]	Specific IgE to Common Allergens >0.35 IU/mL	Number of animal houses with 500 m of the home	≤5	OR	1.00	
			≤10	OR	0.95	0.65–1.39
			≤12	OR	1.38	0.55–3.47
			>12	OR	0.54	0.17–1.69
[62]	Bronchial Hyper-responsiveness to methacholine	How annoyed are you by odor in and around you home?	Not at all	OR	1.00	
			Somewhat	OR	1.21	0.83–1.76
			Moderately	OR	0.92	0.50–1.69
			Strongly	OR	1.12	0.50–2.49
[62]	Bronchial Hyper-responsiveness to methacholine	Number of animal houses with 500 m of the home?	≤5	OR	1.00	
			≤10	OR	0.72	0.47–1.10
			≤12	OR	0.50	0.17–1.49
			>12	OR	0.38	0.11–1.31
[62]	FEV % predicted	How annoyed are you by odor in and around you home?	Not at all	Mean	0.00	
			Somewhat	Mean	−1.5	−4.0–1.0
			Moderately	Mean	0.2	−3.7–4.2
			Strongly	Mean	−0.1	−5.2–5.0
[62]	FEV % predicted	Number of animal houses with 500 m of the home	≤5	Mean	0.00	
			≤10	Mean	−0.1	−2.8–2.6
			≤12	Mean	0.2	−6.9–7.3
			>12	Mean	−7.4	−14.4–0.4
[69]	log salivary IgA concentration (µg/ml).	Odor coded as a seven-level continuous variable (nine-level variable recoded: 1-3, 4, 5, 6, 7, 8, 9). (n = 15)		Beta	−0.058	−0.12–0.004
[69]	log salivary IgA secretion rate (µg/ml)	Odor coded as a seven-level continuous variable (nine-level variable recoded: 1–3, 4, 5, 6, 7, 8, 9). (n = 15)		Beta	−0.054	−0.12–0.012
[65]	Heart rate					p = 0.78
	Respiratory rate					p = 0.57
	Temperature					p = 0.27
	Systolic blood pressure					p = 0.70
	Diastolic blood pressure					p = 0.27
	Blood pressure ratio (systolic to diastolic)					p = 0.52
	Percent change FEV1					p = 0.98
	Percent change FVC					p = 0.80
	Percent change FEF 25–75%					p = 0.88
	Salivary IgA (µg/mL)					p = 0.57
	Digit span score					p = 0.35
	IL-8 (pg/mL)					p = 0.11
	IL-1β (pg/mL)					p = 0.38
	Cell counts					p = 0.76
	Percent epithelial cells			Beta	−21.1	p = 0.02
	Percent lymphocytic cells			Beta	23.0	p = 0.008
	Percent PMNs					p = 0.22
	Absolute epithelial cells					p = 0.15
	Absolute lymphocytic cells					p = 0.78
	Absolute PMNs					p = 0.27

*

[42] Adjusted for gender, oldest sibling, experienced street noise (clearly vs. very little), actual smoking (yes vs. no), education level, breastfed at least 4 months (yes vs. no), mold (yes vs. no), contact with cats at a young age (yes vs. no), rug/Carpeted floor (yes vs. no), parental atopy.

[68] Adjusted for individual-level characteristics (gender, age, race, Hispanic ethnicity, economic status, smoking status, exposure to second-hand smoke at home, and use of a gas stove more than once per month) and school-level characteristics (rural locale, indoor air quality, and reports of other non-livestock industries nearby).

[62] Adjusted for age (5 categories), sex, active and passive smoke exposure, level of education, number of siblings, parental allergies.

[69] Adjusted for fixed effects for odor, time of day, and day, and random effects for cluster, person within cluster, odor, and time of day.

[65] Two-way analysis of variance.

The adjusted associations between self-reported disease outcomes and non-odor related explanatory variables are presented in TABLE 4. In a German study that assessed the variable self-reported wheeze, the odds of disease were highest in German adults living with >12 animals houses within 500 meters (OR = 2.45, 95% CI 1.22 to 4.90) [62]. However, the majority of significant associations were identified when study subjects with allergies or parents with allergies were evaluated. Children with self-reported allergies reported increased prevalence of self-reported wheeze if they lived within two to three miles of the nearest AFO (OR = 1.12, 95% CI 1.04 to 1.19), attended schools with less than two million hog pounds within three miles of the school (OR = 1.07, 95% CI 1.01–1.12) and were in the low exposure category (OR = 1.10, 95% CI, 1.03–1.18). Surprisingly, the categories of these variables representing the highest level of these exposure measures were not associated with increased prevalence of disease, i.e., children with self-reported allergies who lived within two miles of the nearest AFO (OR = 1.01, 95% CI, 0.95–1.07), attended schools with five million hog pounds within three miles of school (OR = 1.00, 95% CI 0.89 to 1.11) and were in the high exposure category (OR = 1.01, 95% CI 0.89 to 1.11). Further, evidence of a dose response gradient was not apparent as the odds ratio for the disease exposure relationship were closer to the null value in high exposure categories compared to the point estimates for the lower exposure categories. It should be noted that no authors used statistical methods to assess a trend for ordinal variables; therefore it is our interpretation that no dose gradient is apparent. Again, the remaining associations did not indicate strong associations as the point estimates were close to the null value and confidence intervals were wide.

**Table 4:** The adjusted* association between the self-reported outcome variables and non-odor related explanatory variables in communities near AFOs.

Study	Outcome variable	Community health/animal exposure measure	Subcategory	Effect measure	Point estimate	95% CI point estimate
[42]	Asthmatic pathology in children with non-atopic parents	Log of endotoxin for each additional	NA	OR	0.95	0.88–1.05
[42]	Asthmatic pathology in children with atopic parents	Log of endotoxin for each additional item	NA	OR	1.15	1.03–1.29
[68]	Self-reported occurrence of wheeze at any time in the past 12 months	Miles to nearest CAFO for children with self-reported allergies	>3	OR	1.00	
			≤3	OR	1.05	1.00–1.10
			2 to ≤3	OR	1.12	1.04–1.19
			≤2	OR	1.01	0.95–1.07
[68]	Self-reported occurrence of wheeze at any time in the past 12 months	Miles to nearest CAFO for children with no self-reported allergies	>3	OR	1.00	
			≤3	OR	1.02	0.94–1.11
			2 to ≤3	OR	1.08	0.95–1.21
			≤2	OR	0.99	0.89–1.09
[68]	Self-reported occurrence of wheeze at any time in the past 12 months	Hog pounds (in millions within 3 miles of school) for children with self-reported allergies	<2.0	OR	1.07	1.01–1.12
			2.0 to <5.0	OR	1.04	0.93–1.14
			≥5.0	OR	1.00	0.89–1.11
[68]	Self-reported occurrence of wheeze at any time in the past 12 months	Hog pounds (in millions within 3 miles of school) for children with no self-reported allergies	<2.0	OR	1.03	0.93–1.12
			2.0 to <5.0	OR	0.99	0.81–1.16
			≥5.0	OR	1.04	0.85–1.23
[68]	Self-reported occurrence of wheeze at any time in the past 12 months	Exposure category for children with self-reported allergies	None	OR	1.00	
			Low	OR	1.10	1.03–1.18
			Medium	OR	1.04	0.97–1.12
			High	OR	1.01	0.89–1.11
[68]	Self-reported occurrence of wheeze at any time in the past 12 months	Exposure category for children with no self-reported allergies	None	OR	1.00	
			Low	OR	1.09	0.95–1.23
			Medium	OR	1.01	0.89–1.13
			High	OR	0.97	0.84–1.23
[62]	Self-reported outcomes: wheeze without a cold in the last 12 months	Number of animal houses with 500 m of the home	≤5	OR	1.00	
			≤10	OR	1.00	0.70–1.42
			≤12	OR	1.62	0.74–3.53
			>12	OR	2.45	1.22–4.90
[62]	Self-reported outcomes: physician diagnosis of asthma (ever?)	Number of animal houses with 500 m of the home	≤5	OR	1.00	
			≤10	OR	0.69	0.42–1.11
			≤12	OR	1.23	0.43–3.54
			>12	OR	1.18	0.45–3.10
[62]	Self-reported outcomes: symptoms of allergic rhinitis	Number of animal houses with 500 m of the home	≤5	OR	1.00	
			≤10	OR	0.91	0.66–1.24
			≤12	OR	1.20	0.56–2.57
			>12	OR	1.29	0.64–2.60
[65]	Headache			OR	4.1	p = 0.001
	Sore throat					p = 0.27
	Itchy throat					p = 0.12
	Eyes irritated			OR	6.1	p = 0.004
	Eyes tearing	Model didn't converge				
	Nasal congestion					p = 0.76
	Nasal secretion					p = 0.22
	Nasal irritation					p = 0.34
	Difficulty breathing	Model didn't converge				
	Cough					p = 0.66
	Nausea			OR	7.8	p = 0.014

*

[42] Adjusted for gender, oldest sibling, experienced street noise (clearly vs. very little), actual smoking (yes vs. no), education level, breastfed at least 4 months (yes vs. no), mold (yes vs. no), contact with cats at a young age (yes vs. no), rug/Carpeted floor (yes vs. no), parental atopy.

[68] Adjusted for individual-level characteristics (gender, age, race, Hispanic ethnicity, economic status, smoking status, exposure to second-hand smoke at home, and use of a gas stove more than once per month) and school-level characteristics (rural locale, indoor air quality, and reports of other non-livestock industries nearby).

[62] Adjusted for age (5 categories), sex, active and passive smoke exposure, level of education, number of siblings, parental allergies.

[69] Adjusted for fixed effects for odor, time of day, and day, and random effects for cluster, person within cluster, odor, and time of day.

[65] Two-way analysis of variance.

Studies using self-reported measures of disease occurrence and odor as a measure of exposure to AFO are presented in TABLE 5. There was a consistent strong association between self-reported disease and the highest level of odor. For these associations there was a indication of a dose gradient, as the point estimate for the odds ratio increased as the level of annoyance or odor detection increased for four of the five associations evaluated. However again, the presence of a dose gradient was not evaluated formally. When the association between clinical outcomes and odor exposure were evaluated, the associations were weaker and less inconsistent (TABLE 3). The reporting by the authors of p values rather than effect sizes hampered the full interpretation of these associations.

**Table 5:** The adjusted* association between the self-reported health outcomes and odor measures in communities near AFOs.

Study	Outcome variable	Community health/animal exposure measure	Subcategory	Effect measure	Point estimate	95% CI point estimate
[68]	Self-reported occurrence of wheeze at any time in the past 12 months	Livestock odor for children with self-reported allergies	Outside school only	OR	1.04	0.98–1.09
			Outside + inside <2 times/month	OR	0.99	0.93–1.06
			Outside + inside ≥2 times/month	OR	1.24	1.03–1.44
[68]	Self-reported occurrence of wheeze at any time in the past 12 months	Livestock odor for children with no self-reported allergies	Outside school only	OR	0.94	0.85–1.02
			Outside + inside <2 times/month	OR	1.04	0.93–1.15
			Outside + inside ≥2 times/month	OR	1.21	0.85–1.57
[62]	Self-reported outcomes: wheeze without a cold in the last 12 months	How annoyed are you by odor in and around you home?	Not at all	OR	1.00	
			Somewhat	OR	1.23	0.90–1.68
			Moderately	OR	2.19	1.42–3.37
			Strongly	OR	2.96	1.80–4.86
[62]	Self-reported outcomes: physician diagnosis of asthma (ever?)	How annoyed are you by odor in and around you home?	Not at all	OR	1.00	
			Somewhat	OR	1.40	0.95–2.06
			Moderately	OR	1.51	0.84–2.73
			Strongly	OR	2.51	1.32–4.75
[62]	Self-reported outcomes: symptoms of allergic rhinitis	How annoyed are you by odor in and around you home?	Not at all	OR	1.00	
			Somewhat	OR	1.09	0.83–1.42
			Moderately	OR	1.49	1.00–2.22
			Strongly	OR	1.81	1.11–2.97

*

[68] Adjusted for individual-level characteristics (gender, age, race, Hispanic ethnicity, economic status, smoking status, exposure to second-hand smoke at home, and use of a gas stove more than once per month) and school-level characteristics (rural locale, indoor air quality, and reports of other non-livestock industries nearby).

[62] Adjusted for age (5 categories), sex, active and passive smoke exposure, level of education, number of siblings, parental allergies.

## Discussion

The purpose of this review was to evaluate the studies reporting the association between animal feeding operations and measures of the health of individuals living near animal feeding operations but not actively engaged in livestock production in North America, the European Union, the United Kingdom, and Scandinavian countries. Based on the magnitude and the consistency of associations observed there was little compelling evidence for a consistent strong association between clinical measures of disease and proximity to AFOs. However, the body of work is small in this area and based on epidemiological studies which have greater potential for bias.

There was inconsistent evidence of a small increase in self-reported disease in people with allergies or familial history of allergies. The magnitude of associations for this subgroup of the population lay within 10% points of the null value (0.99 to 1.12) indicating a<10% increase in the prevalence of adverse health outcomes, with one exception, which reported an approximately 20% increase in prevalence of adverse outcomes. What was surprising about these associations was the lack of any indication of a dose response. Evidence of a dose response would have added weight to evidence of an association. For all of the associations evaluated, the explanatory variables were ordinal in nature, presumably designed to capture a dose response. The WHO Guidelines for Evaluation of Environmental Evidence suggest that the presence of a biological gradient is helpful in proposing a causal association for environmental health hazards [18].

There was evidence of a dose response for exposure variables that described aversion to odor; those individuals with the strongest aversion/detection to livestock odor were associated with the highest odds of self-reported wheeze. Using the odds ratio as the effect measure, the magnitude of the associations with odor were high, up to 300% increase in the odds of self reported outcomes in individuals who were strongly annoyed by odor. However, none of the clinical measures showed an association with measures of odor, which would have made the associations more compelling and demonstrated consistency of the association across various outcome measures. The location of the effect measure estimates and the width of the corresponding confidence intervals for clinical measure of disease showed little evidence of a consistent association, even a weak association, across the studies.

In an effort to understand how to establish causation claims when evaluating environmental causes, the report, “ Identifying the environmental causes of disease: how should we decide what to believe and when to take action,” published by The Academy of Medical Sciences, discussed several examples where non-experimental evidence has been used to evaluate environmental causes of disease [71]. The report discussed examples where non-experimental evidence had led to relatively strong inferences, where non-experimental research had led to cases with probably valid causal inference, and where non-experimental research had led to probably misleading causal claims. Many conclusions and recommendations were included in the report, however relevant to this review was the observation that examples where non-experimental evidence had led to relatively strong inferences, shared several common features including a very large effect (such as lung cancer and smoking) or they applied to rare or unusual outcomes with distinctive features (neural tube defects and folate deficiency). Other characteristics were attention to alternative explanations and the availability of many studies conducted in multiple populations [71]. Based on these observations, the body of evidence in this review is likely inadequate to evaluate causation because evidence is available from very few studies and the disease outcomes evaluated tend to be common non-specific outcomes, i.e., self reported wheeze.

A previous narrative review of the topic has suggested that “sufficient research supports actions to protect rural residents from the negative impacts of CAFOs on community health” and only mechanism research was warranted [7]. However, the results of the current review do not strongly support this statement. The results of this review suggest that further research is warranted, particularly toward understanding proximity to animal agriculture, odor and mental health and the subgroup of people with self-reported allergies [7].

Several expert committee reports have provided guidelines on how to assess an association between an environmental exposure and disease occurrence [18], [71]. Both reports recommended that sources of bias be considered in primary research before concluding that causal associations exist. Based on our evaluation, we propose that the studies in this body of work could be viewed as two groups of work. The first group consisted of studies of greater use for establishing causation because of the design and execution of the study, of these there are currently too few, however they represent the majority of studies in this body of work (5 of 9). The second group consisted of studies that might be considered of less value for establishing causation and better for hypothesis generation because of the study design or execution.

It is imperative that future researchers evaluate the characteristics of the studies in the body of work and understand the limitations and strive to improve the designs used. Such an approach to future research will improve the evidentiary value of the work and its use for decision-making. Recommendations for design features that should be incorporated into future studies would include the use of quantifiable clinical outcomes and measures of exposure to AFOs, limits on the number of outcomes assessed or adjustment for multiple comparisons, inclusion of sample size justification and the null hypothesis to be tested, random selection of study participants, longitudinal study designs, appropriate evaluation of dose responses and the use of statistical methods that account for clustering when appropriate. Further, the combination of experimental and observational studies will likely be helpful in future causal discussions. Both study types should be included in future research as evidence from a mixture of well executed studies will be important for establishing if a causal association exists. This characteristic was a hallmark of prior examples where non-experimental evidence led to strong causal inference i.e. “In no instance, did one design provide the ‘clinching’ proof, but, in combination, they made causal inference a compelling probability“ [71].

Another recommendation is recognizing the hypothesis generating nature of some of the studies in the body of work. The concept that all research is not of equal evidentiary evidence value is not a new one and is the basis of the evidence pyramid [72]. The area of environmental health assessment represents one of the areas where reliance of the epidemiological studies is often necessary; however even within epidemiological studies it is possible to assess internal validity. Included within this group of hypothesis generating studies are the ecological studies, which report associations between animal density and the occurrence of disease. Due to the potential for ecological fallacy, these studies should not be used for causal inference although some articles do seem to draw causal conclusions from the study results.

We encourage readers to evaluate the rationale for the discussion of biases within the studies as these represent a critical component – transparency – of the systematic review methodology. Finally, as systematic reviews place a heavy emphasis on transparency, it should be noted that several of the panel members have previously authored narrative reviews of this topic [9], [14] and two members of the review panel have previously received research funding for unrelated areas of swine health by the National Pork Board, which also funded this review. Four panel members have served as an advisory meeting members or as grant reviewers for the National Pork Board research program in the past 10 years.
